# Cultivation in a Natural Microbial Community Enhances the Industrial Performance of a Genetically Engineered Cyanobacterium for Bioplastic Production

**DOI:** 10.1111/1751-7915.70302

**Published:** 2026-01-20

**Authors:** Arianna Zini, Jennifer Müller, Phillipp Fink, Karl Forchhammer

**Affiliations:** ^1^ Interfaculty Institute for Microbiology and Infection Medicine Tübingen, Organismic Interactions Department University of Tübingen Tübingen Germany; ^2^ Institute of Medical Microbiology and Hygiene University of Tübingen Tübingen Germany; ^3^ Core Facility Genomics, Medical Faculty University Hospital Tübingen/DFG‐Funded NGS Competence Center Tübingen Tübingen Germany

**Keywords:** Engineered cyanobacteria, Hybrid microbiomes, Photobioreactors, Polyhydroxybutyrate (PHB)

## Abstract

Large‐scale production of polyhydroxybutyrate (PHB), a biodegradable bioplastic, using genetically engineered cyanobacteria offers a sustainable alternative to petrochemical‐derived plastics. However, monoculture‐based phototrophic systems face major limitations, such as poor resilience in large‐scale reactors, hindering industrial upscaling. To address these challenges, we replaced the native cyanobacterium of a natural microbial consortium with a genetically engineered *Synechocystis* strain optimised for PHB production, establishing what we define a hybrid photosynthetic microbiome. This new community preserved the ecological structure and stability of the original microbiome while gaining synthetic production capacity. Compared to the axenic strain, the hybrid system exhibited enhanced robustness under abiotic stress, including light and temperature fluctuations, and improved tolerance to operational instability. These features made it suitable for upscaling and application in non‐sterile environments. The hybrid microbiome sustained PHB production in scaled photobioreactors, reaching up to 32% PHB per cell dry weight (CDW) equal to ~230 mg L^−1^ under fully photoautotrophic conditions. Production was also achieved under dark conditions with acetate supplementation, highlighting the system's metabolic flexibility. This work demonstrates the successful integration of an engineered phototroph into a stable native microbiome, positioning hybrid communities as powerful platform for industrial biotechnology.

## Graphical abstract

We developed a hybrid photosynthetic microbiome, in which the native cyanobacterium of a natural microbial consortium was replaced with a genetically engineered *Synechocystis* strain optimised for polyhydroxybutyrate (PHB) production. The hybrid system demonstrated improved tolerance to abiotic stresses, making it suitable for upscaling.

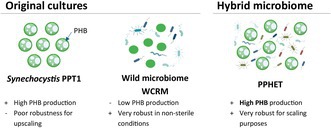

## 1 Introduction

Polyhydroxybutyrate (PHB), a member of the polyhydroxyalkanoate (PHA) family, is a biodegradable polymer synthesised by various bacteria and archaea as an intracellular carbon and energy storage under nutrient‐limited conditions (Gundlapalli and Ganesan 2025; McAdam et al. 2020). Its favourable mechanical properties, combined with its biodegradability in biologically active environments, make PHB a promising substitute for petrochemical‐based plastics (Gautam et al. 2024; McAdam et al. 2020).

*Synechocystis* sp. PCC 6803 (*Synechocystis* from now on) is a well‐established model cyanobacterium and a natural PHB producer. Under nitrogen limitation, *Synechocystis* undergoes chlorosis, a dormant state characterised by degradation of the photosynthetic apparatus and accumulation of storage carbohydrates, namely glycogen and PHB (Doello et al. 2021; Forchhammer and Schwarz 2019). Leveraging this, in previous work, we characterised and engineered the metabolic pathways sustaining PHB synthesis (Orthwein et al. 2021). This resulted in generation of a new strain termed *Synechocystis* sp. PCC 6803 PPT1 (PPT1 from now on), capable of accumulating up to 60% PHB per cell dry weight (CDW) during nitrogen‐starvation when NaHCO_3_ was used as a carbon source, and 80% when sodium acetate was used (Koch et al. 2020). However, due to the low biomass in the cultures, overall productivity was limited to 5 mg L^−1^ day^−1^.

A key limitation to the scalability of cyanobacteria are the high production costs, driven by low product yields, expensive equipment and significant maintenance requirements (Price et al. 2022; Rueda et al. 2023). Moreover, in large, high‐density cultures, limited light penetration hampers the onset of chlorosis, making PHB accumulation difficult even after complete nitrate consumption (Klotz et al. 2015). Monocultures are sensitive to environmental stresses common in industrial‐scale cultivation, such as fluctuating light intensity, temperature shifts, mechanical disturbances and occasional invasion by foreign microorganisms that outcompete cyanobacteria (Awasthi et al. 2014; Huang et al. 2017). These challenges add on top of the primary limitation associated with cyanobacterial scalability: high production costs.

Diverse microbial communities are generally more resilient to environmental stress and support broader metabolic functionality than monocultures (Awasthi et al. 2014; Jousset et al. 2011). Therefore, synthetic and engineered consortia are emerging as valuable tools in industrial biotechnology, offering improved adaptability and productivity (Giri et al. 2020). Cyanobacteria‐enriched microbiomes derived from natural environments have demonstrated robustness under non‐sterile conditions in pre‐industrial photobioreactors, making them a cost‐effective platform for large‐scale applications (Altamira‐Algarra et al. 2024).

We hypothesised that replacing the native phototroph in a stable, natural microbiome with a genetically engineered cyanobacterium could overcome the limitations of axenic cultures. To test this, we introduced the strain PPT1 into a protective microbial community, creating what we term a *hybrid microbiome*: a native microbial consortium in which certain species have been selectively substituted with engineered ones. This strategy preserves the ecological integrity of the original community while enabling new synthetic functions, offering a robust platform for advancing phototrophic biotechnologies.

## 2 Experimental Procedures

### 2.1 Cyanobacterial Cultivation Conditions

All cyanobacterial cultures, axenic and community‐based, were pre‐cultured in BG11 medium supplemented with 5 mM NaHCO_3_ under continuous shaking (120 rpm) at 28°C and light intensity of ~50–60 μmol m^−2^ s^−1^ (Rippka et al. 1979). Antibiotics (kanamycin and spectinomycin, 50 μg mL^−1^ each, depending on the strain's resistance) were included for axenic cultivation of mutant strains but omitted in microbiome cultures. Pre‐cultures at OD_750_~1 were harvested by centrifugation (15 min, 4200 rpm) and resuspended in BG11_0_ medium (BG11 lacking NaNO_3_). The traditional two‐stage PHB production process, comprising an initial growth phase in BG11 followed by re‐inoculation of the pellet into BG11_0_, was replaced with a simplified one‐step protocol. Cultures were directly inoculated into BG11_0_ supplemented with 4–8 mM NaNO_3_ and NaHCO_3_ as a carbon source at a starting OD_750_ of 0.2. As nitrate became limiting, cells transitioned into chlorosis and initiated PHB biosynthesis, thus allowing the need for medium exchange to be circumvented. Small‐scale experiments were conducted in 100 mL Erlenmeyer flasks containing 50 mL of culture.

For large‐scale setups, two types of reactors were used. In 12 L borosilicate glass vessels (cultivation volume: 8–10 L), mixing was provided with a magnetic stirrer, supplemented by an air dispenser positioned at the middle of the culture to enhance circulation (air dispenser not used for the dark fermentation approach). In custom flat‐panel reactors (dimensions: ~50 × 26 × 3 cm; volume: 4 L), mixing was provided by a magnetic stirrer located at the centre of the reactor. Sterile conditions involved standard aseptic techniques, including the use of autoclaved media and laminar flow handling. Non‐sterile conditions were mimicked by inoculating cultures in ambient air using non‐autoclaved BG11 medium. For cultivation on solid medium, cultures were grown on BG11 Difco‐agar 1.5% supplemented with 5 mM NaHCO_3_ to assess colony formation, or on BG11 supplemented with glucose and casamino acids for contamination screening.

A list of the strains and microbial communities used in this study is provided in the Table S1.

### 2.2 Selective Isolation of Heterotrophic Community Members

**FIGURE 1:**
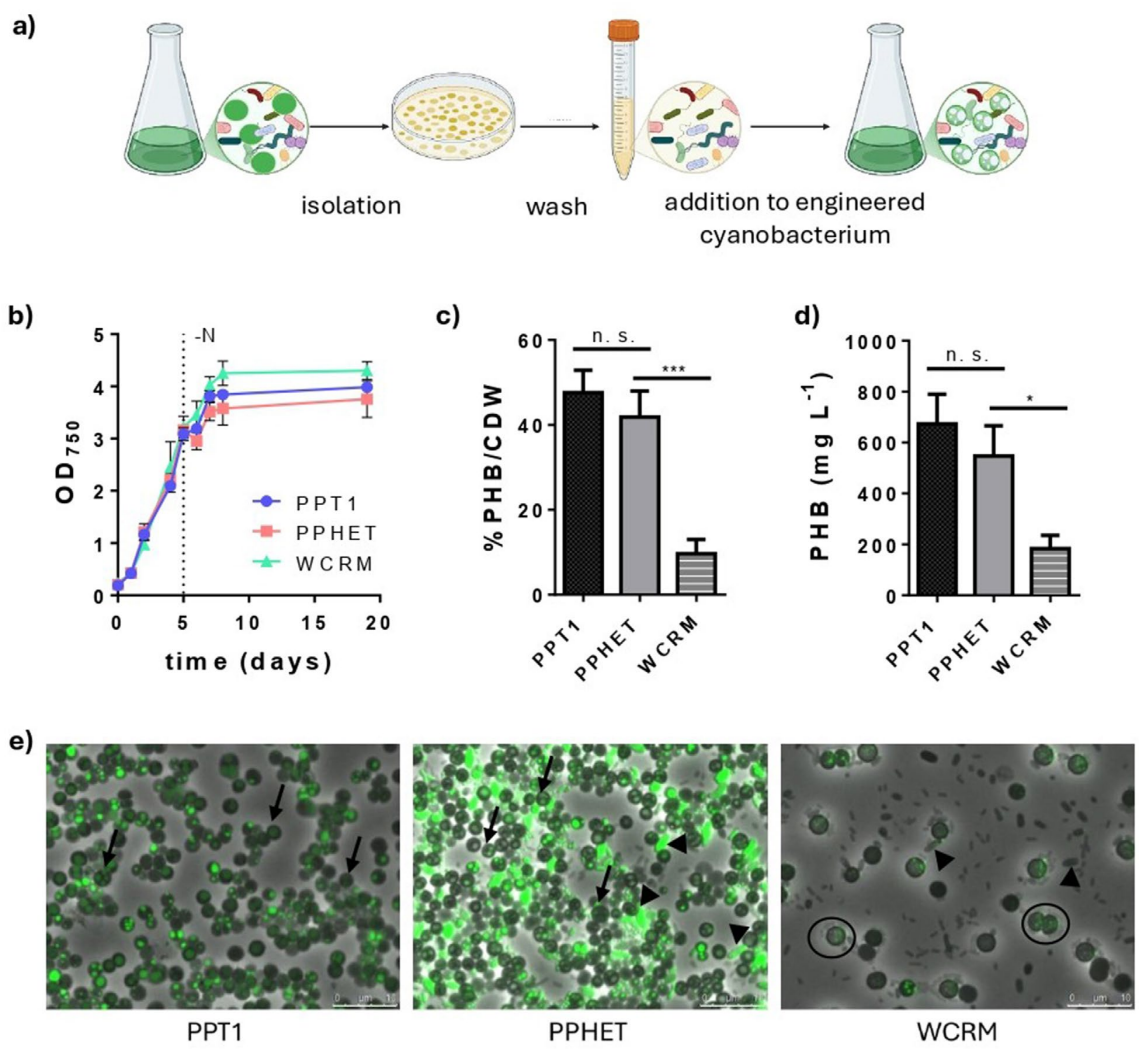
Generation and growth of hybrid cyanobacterial microbiomes. (a) Schematic representation of the method used to generate hybrid microbiomes by replacing the native cyanobacterial component of the wild‐type community with a genetically engineered strain. (b) Growth curve of PPT1 (blue), PPHET (pink) and a wild cyanobacteria‐rich microbiome (WCRM, green) over 19 days in a one‐step cultivation PHB production process. The dotted line represents the point at which nitrate (NO_3_^−^) had been totally consumed; (c and d) PHB production of PPT1, PPHET and WCRM at the end of the process in terms of %PHB/CDW (c) and in mg L^−1^ (d); data points and bar columns represent the mean of three biological replicates at each time point ±SD. For the PHB evaluation, statistical significance was determined using one‐way ANOVA with Brown‐Forsythe test for variance and Dunnett's multiple comparisons test against the control group (PPHET). (e) Microscopic pictures of PPT1, PPHET and WCRM at the end of the process, following staining of PHB granules with BODIPY (green). Images show overlays of phase‐contrast and GFP fluorescence channels. Arrows indicate PPT1 cells with PHB stained granules in the axenic (PPT1) and the hybrid microbiome (PPHET) culture. Arrowheads indicate non‐*Synechocystis* members of the community in PPHET and WCRM which display stained granules. Circles indicate the wild *Synechocystis* originally present in the natural community. Images show one representative of the three biological replicates per condition.

The Wild Cyanobacteria‐Rich Microbiome (WCRM) we used, previously described (Altamira‐Algarra et al. 2024), is characterised by the presence of diverse cyanobacterial taxa, most of which belonged to the *Synechocystis* sp. To enrich and isolate the heterotrophic fraction of the WCRM, cultures were plated on BG11 prepared with agar‐agar (Bernd Euler Biotechnologie‐Mikrobiologie), instead of Difco‐agar. Plates were incubated at 28°C under dim light (10–20 μmol m^−2^ s^−1^) for 2–4 days (Figure 1a). This approach enabled the selective proliferation of the non‐cyanobacterial part of the community. Growth was supported by residual organic compounds from the inoculum, derived from cyanobacterial exudates during prior liquid cultivation. For qualitative assessment of heterotrophic diversity and abundance, culture aliquots were also plated on LB agar (Luria Bertani, Lennox).

### 2.3 Microscopy and Staining Procedure

Fluorescence microscopy was performed on a LeicaDM5500B (Leica Microsystems, Germany) with objective lenses and filter cubes. PHB granules were visualised via BODIPY staining using the GFP channel, with a modified method previously described (Fink et al. 2025). Briefly, 1.5 μL of BODIPY dye solution (1 mg mL^−1^ in DMSO) was added to 100 μL of cyanobacterial culture. After 10 min of incubation in the dark at room temperature, cells were washed two times with phosphate‐buffered saline (PBS) and then applied on microscope slides pre‐coated with 1% (w/v) agarose to immobilise cells for imaging.

### 2.4 Light Intensity Measurement

Light intensity was measured using a factory‐calibrated photometer from LI‐COR, Model LI‐189. Instrument performance was routinely assessed through repeated measurements under identical conditions.

### 2.5 Chlorophyll *a* Quantification

Chlorophyll *a* (Chl *a*) content was quantified using methanol extraction. A 1 mL culture sample was centrifuged at 14,000 rpm for 5 min, and the supernatant was discarded. The pellet was resuspended in 1 mL of 90% methanol and incubated in the dark at 4°C for 30 min to allow pigment extraction. After incubation, samples were centrifuged again at 14,000 rpm for 5 min, and the absorbance (A) of the supernatant was measured at 665 nm using 90% methanol as a blank. Chlorophyll *a* concentration was calculated using the following equation:

$$\mathrm{Chl}\;a\;\left(\mathrm{\mu g}/\mathrm{mL}\right)=A_{665}\times 13.9.$$

### 2.6 Nitrate (NO_3_ ^−^) Determination

Nitrate concentrations were qualitatively monitored using semi‐quantitative test strips (QUANTOFIX Nitrate/Nitrite, Macherey‐Nagel GmbH & Co. KG, Düren, Germany). These strips provide a detection range of 10–500 mg L^−1^ for NO_3_^−^ and 1–80 mg L^−1^ for NO_2_^−^. Measurements were used to approximately estimate nitrogen depletion during cultivation.

### 2.7 pH Measurements

The pH of culture samples was measured using a Mettler Toledo FiveEasy pH/mV metre equipped with an LE422 microelectrode. The electrode has a measurement range of pH 0–14 and is suitable for temperatures up to 80°C.

### 2.8 O_2_ Measurement

O_2_ saturation was measured with the Hamilton VisiFerm RS485‐ECS 120 sensor according to the manufacturer instructions.

### 2.9 PHB Quantification

The method utilised was previously described (Fink et al. 2025). Shortly, 5–10 mL bacterial culture was harvested, cell dry weight was determined using the same protocol for both axenic and mixed microbial cultures and subsequently boiled in 1 mL of concentrated H_2_SO_4_ (18 M) for 1 h at 100°C to convert PHB to crotonic acid. The boiled cell culture was diluted 1:20 with 0.014 M H_2_SO_4_ and centrifuged at 20,000 *g* for 5 min at RT. The supernatant was analysed by HPLC (HITACHI Chromaster, VWR, Germany) using a Nucleosil 100–5 C18 reverse‐phase column (125 × 3 mm, 5 μm, 100 Å) fitted with a precolumn (4 × 3 mm). Samples (5 μL) were eluted isocratically at 1 mL/min using 30:70 MeOH/20 mM phosphate buffer (pH 2.5) for 10 min, and crotonic acid was detected at 210 nm. PHB standards and crotonic acid calibration curves were used for quantification via linear regression.

### 2.10 16S Library Preparation and Sequence Analysis

Samples were collected from the respective cultures 1 week after inoculation in full BG11. Sample preparation and sequencing was done at Core Facility Genomics/NCCT Microbiology, Institute for Medical Microbiology and Hygiene, University Hospital Tübingen. Genomic DNA was isolated using the Quick DNA Fungal/Bacterial Miniprep Kit (Zymo Research) according to the manufacturer's protocol, with 10 min bead beating in ZR BashingBead Lysis Tube (Zymo Research). The resulting DNA was quantified with a Qubit dsDNA BR/HS Assay Kit (Thermo Fisher). Libraries for 16S amplicon sequencing were prepared in a two‐step PCR approach: using KAPA HiFi HotStart ReadyMix (Roche), primers 515F and 806R (Caporaso et al. 2011) (~350 bp fragment of the 16S V4 region), and index primer mix (IDT for Illumina DNA/RNA UD Indexes, Tagmentation). The pool was sequenced on an Illumina MiSeq device using the MiSeq Reagent Nano Kit v2 (500 cycles) with 2 × 250 bp read length and a depth > 100 k reads per sample.

Data were demultiplexed with the nf‐core/demultiplex v1.4.1 pipeline (Ewels et al. 2020) and further processed with the nf‐core/ampliseq v2.11.0 pipeline (Straub et al. 2020) of the nf‐core collection of workflows (Ewels et al. 2022). Both pipelines use reproducible software enviroments from Bioconda (Grüning et al. 2018) and Biocontainers projects (Da Veiga Leprevost et al. 2017).

Data quality was evaluated, and all primer sequences were trimmed. Adapter and primer‐free sequences were processed sample‐wise (independent) with DADA2 v1.30.0 (Callahan et al. 2016). Taxonomic classification was performed by DADA2 and the database ‘Silva 138.1 prokaryotic SSU’ (Quast et al. 2012). ASV (Amplicon Sequence Variants) sequences, abundance and DADA2 taxonomic assignments were loaded into QIIME2 v2023.7.0 (Bolyen et al. 2019).

## 3 Statistical Analysis

Small‐scale experiments were performed in two or three biological replicates, as indicated in the corresponding figures or sections. Experiments conducted in duplicate at small scale were qualitatively validated through repetition in upscaled systems to assess consistency in system performance. Data are presented as mean values, with error bars representing the standard deviation (±SD). A *p*‐value of less than 0.05 (*p* < 0.05) was considered statistically significant. Statistical analyses were performed using GraphPad Prism version 6. Depending on the experimental design, either one‐way or two‐way ANOVA was applied. For one‐way ANOVA, data distributions were assessed by the software and confirmed to be normal (Gaussian), while equality of variances was confirmed using the Brown‐Forsythe test, followed by Dunnett's multiple comparisons test to compare experimental groups to a control. For two‐way ANOVA, sphericity was assumed as implemented in the software and Sidak's multiple comparisons test was used to evaluate differences between groups.

## 4 Results

### 4.1 Generation of the Hybrid Microbiome PPHET With the Cyanobacterium PPT1

To construct a stable microbiome, we combined the genetically engineered cyanobacterial strain *Synechocystis* sp. PCC 6803 PPT1 (hereafter PPT1) with the non‐cyanobacterial fraction of a wild cyanobacteria‐rich microbiome (WCRM), previously described in Altamira‐Algarra et al. (2024). After the isolation of the non‐cyanobacterial fraction from WCRM (heterotrophs, hereafter), colonies were resuspended in BG11, serially diluted (*het A* to *het D*), and used to inoculate exponentially growing axenic PPT1 cultures (Figure 1a; Figure S1a). We refer to the new hybrid culture with PPT1 as core species as PPHET consortia. As a control, *Synechocystis* sp. PCC 6803 expressing eGFP (referred to as GFP) (Orthwein et al. 2021) was used in a parallel setup to assess the effect of replacing native cyanobacteria and to validate the engineering approach. No significant differences were observed between growth in communities and in isolation for both PPT1 and GFP (Figure S1a), indicating that the hybrid system did not impair cyanobacterial growth. Chlorophyll *a* content was also quantified and found to be comparable across all conditions, further confirming active phototrophic growth (Figure S1b). Microscopy analysis validated the successful colonisation and replacement of the original phototrophic strain with PPT1 or GFP (Figure S1c). Despite variations in the density of the initial heterotrophic inoculum, the resulting communities converged towards similar structural compositions over time, as evidenced by consistent colonies morphology and abundance on LB agar plates (Figure S1d), suggesting that the metabolic interactions with the cyanobacterium shape the community composition. To confirm the stable integration of PPT1 within the hybrid microbiome, we performed multiple assessments, including culture plating on selective media, repeated propagation cycles, and colony PCR. These results are presented in the Figure S1d,e.

### 4.2 Comparative Analysis of Growth and PHB Accumulation in PPT1, PPHET and WCRM

To evaluate growth dynamics and PHB production, we compared PPT1, PPHET and WCRM. In this experiment, cultures were inoculated in BG11_0_ medium supplemented with 5 mM of NaNO_3_ and 10 mM of NaHCO_3_. Upon nitrogen depletion, 50 mM of NaHCO_3_ were added, to boost PHB accumulation. The experiment was conducted in three biological replicates over 19 days, comprising the initial growth phase followed by chlorosis and PHB accumulation. The final biomass accumulation was comparable in all three systems, with OD_750_ values reaching approximately 4 (Figure 1b). PHB accumulation was assessed both quantitatively and qualitatively. PPT1 accumulated 50% PHB per cell dry weight (CDW). PPHET reached similar PHB levels, up to 48% PHB/CDW, whereas the WCRM system, comprising wild‐type *Synechocystis*, produced around 10% PHB/CDW (Figure 1c). PHB titers and productivity were also evaluated, with PPT1 and PPHET achieving similar performances exceeding 600 mg L^−1^ (Figure 1d), corresponding to a productivity of approximately 35 mg L^−1^ day^−1^.

Fluorescence microscopy with BODIPY staining enabled visualisation of intracellular PHB granules (Figure 1e). Comparable PHB accumulation was observed in PPT1 under axenic and community conditions (indicated by black arrows). Interestingly, in PPHET not only PPT1 but also other members of the community stained positive for BODIPY, suggesting they may be able to accumulate PHB as well (indicated by black arrowheads in PPHET and WCRM). As expected, wild‐type *Synechocystis* in WCRM accumulated fewer and smaller PHB granules (indicated by a black circle). It is also worth noting that non‐*Synechocystis* members of the community in PPHET accumulated more PHB than in WCRM, suggesting that different interactions may occur in the wild microbiome compared to the hybrid one. Additionally, microscopy analysis revealed that wild‐type *Synechocystis* cells in WCRM are larger than the engineered PPT1 cells, offering a visual mean to distinguish the engineered strain from the wild‐type within mixed communities.

### 4.3 Microbial Dynamics and Ecological Insights From 16S rRNA Sequencing

To assess microbial diversity in PPHET compared to WCRM microbiomes, samples were collected 1 week after inoculation in BG11 and standard growing conditions for 16S rRNA amplicon sequencing. The evaluation of data quality revealed that 93.3% of the sequences per sample passed the filtering. After denoising and removal of chimeric reads between 70.88% and 87.19% reads per sample (average 82.5%) were used to determine the amplicon sequencing variants (ASVs). 139 amplicon sequencing variants (ASVs) were obtained across all samples.

**FIGURE 2:**
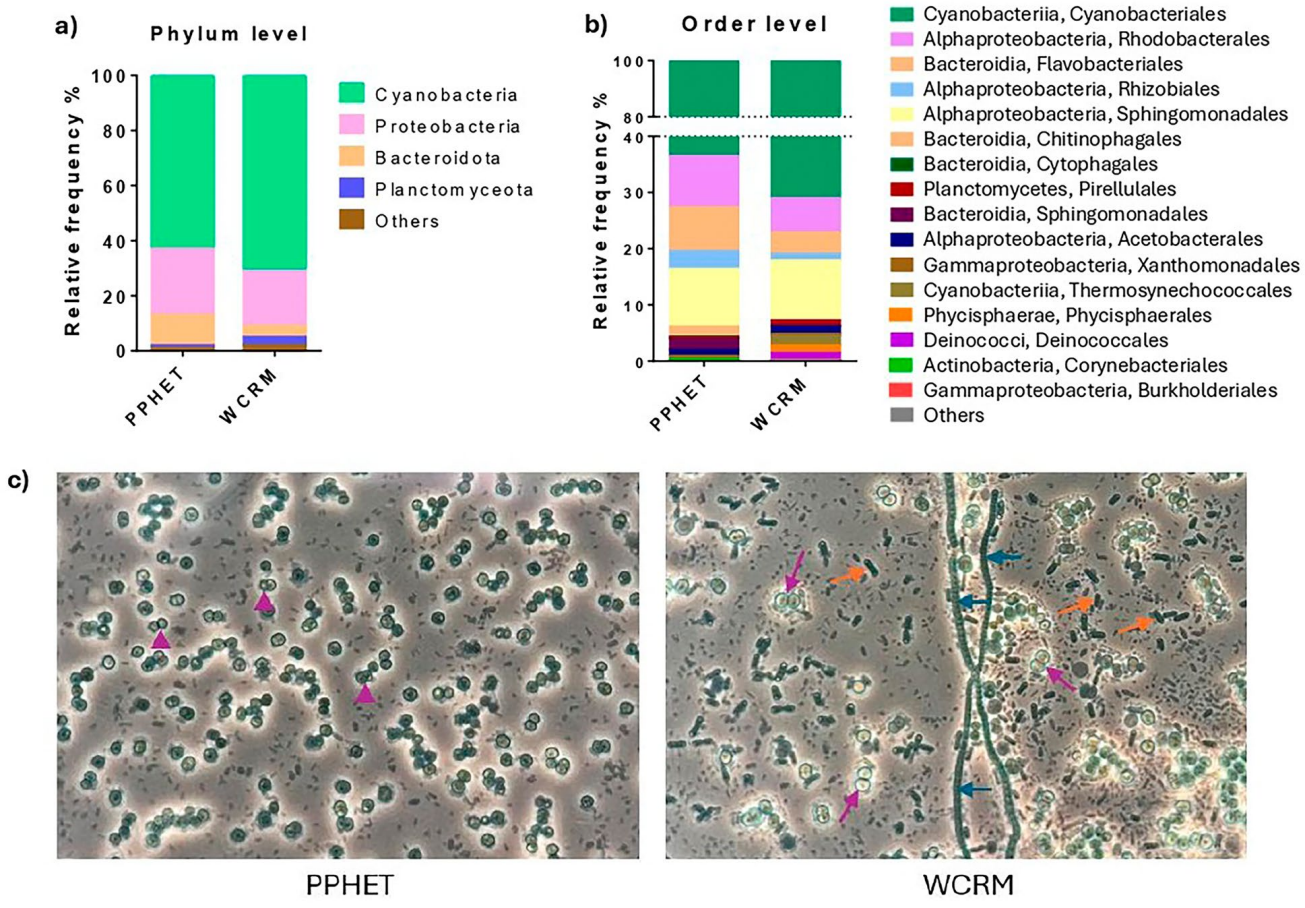
Microbial Community Composition of PPHET and WCRM 7 days after inoculation in BG11. (a and b) Comparison of the biodiversity composition in PPHET and WCRM at the phylum level (a) and at the order level (b). Stacked bar plots show the relative abundance of taxonomic groups within each community. Each bar represents one sample, with segments indicating the proportion of each group. (c) Microscopic pictures of PPHET and WCRM during normal growth in standard BG11. Samples were imaged using transmitted light brightfield microscopy and acquired via eyepiece projection. Purple arrowheads indicate *Synechocystis* PPT1, which appears smaller than *Synechocystis* from the WCRM (purple arrows). In WCRM, orange arrows indicate members of the Thermosynechococcales order, whereas blue arrows indicate filamentous cyanobacteria, likely members of the Phormidesmiales or of the Leptolyngobyales order according to our 16S rRNA results. Images show one representative per sample.

Overall, the two communities shared 80%–90% of their taxonomic composition. At the phylum level, both were dominated by Cyanobacteria, Proteobacteria, and Bacteroidota, with Cyanobacteria representing ~70% of the total community (Figure 2a). The predominant classes included Cyanobacteriia, Alphaproteobacteria, and Bacteroidia. Using the revised cyanobacterial taxonomy according to whole‐genome phylogenomic analyses (Salazar et al. 2020), we performed order‐level taxonomy analysis, which revealed key differences between the two communities. While both PPHET and WCRM contained Cyanobacteriales, reflecting the presence of *Synechocystis*, WCRM also included additional cyanobacterial orders, such as the unicellular Thermosynechococcales and in minor abundancy the filamentous Phormidesmiales and Leptolyngbyales (Table S2), suggesting a multi‐phototrophic community. In contrast, PPHET contained only Cyanobacteriales, consistent with the introduction of a single engineered strain (PPT1) (Figure 2b). Microscopic observations further supported this finding, confirming PPT1 as the dominant, and likely sole, cyanobacterial species in PPHET (blue arrowheads, Figure 2c; black arrows, Figure 1e). These analyses confirm the effectiveness of our approach to selectively retain heterotrophs during community assembly. Despite the overall compositional similarity, ~10% of the community structure differed between WCRM and PPHET. WCRM exhibited higher abundancy in Pirellulales, Acetobacterales, Phycisphaerales, Deinococcales, and Burkholderiales, whereas PPHET was more abundant in Chitinophagales, Cytophagales, and Sphingobacteriales (Figure 2b). Groups of microorganisms with a relative abundancy lower than 0.05% were grouped in “Others” and can be found in the Table S2. These shifts likely reflect selective pressures imposed by the presence of the engineered cyanobacterium.

Even though these results give insights into the ecological composition of PPHET and WCRM, further analysis is needed to better understand community dynamics during the production process and in large‐volume bioreactors for industrial applications.

### 4.4 Robustness of the Hybrid Microbiome Under Biotic and Abiotic Stress Factors

To evaluate the robustness of PPHET compared to the axenic strain PPT1, both systems were tested under various biotic and abiotic stress conditions, including non‐sterile environments, elevated light intensities and temperature fluctuations. Under non‐sterile conditions, despite the moderate reduction in PHB content, both PPHET and PPT1 maintained growth and PHB accumulation, suggesting that this condition plays a minor role in small‐scale systems (Figure S2).

**FIGURE 3:**
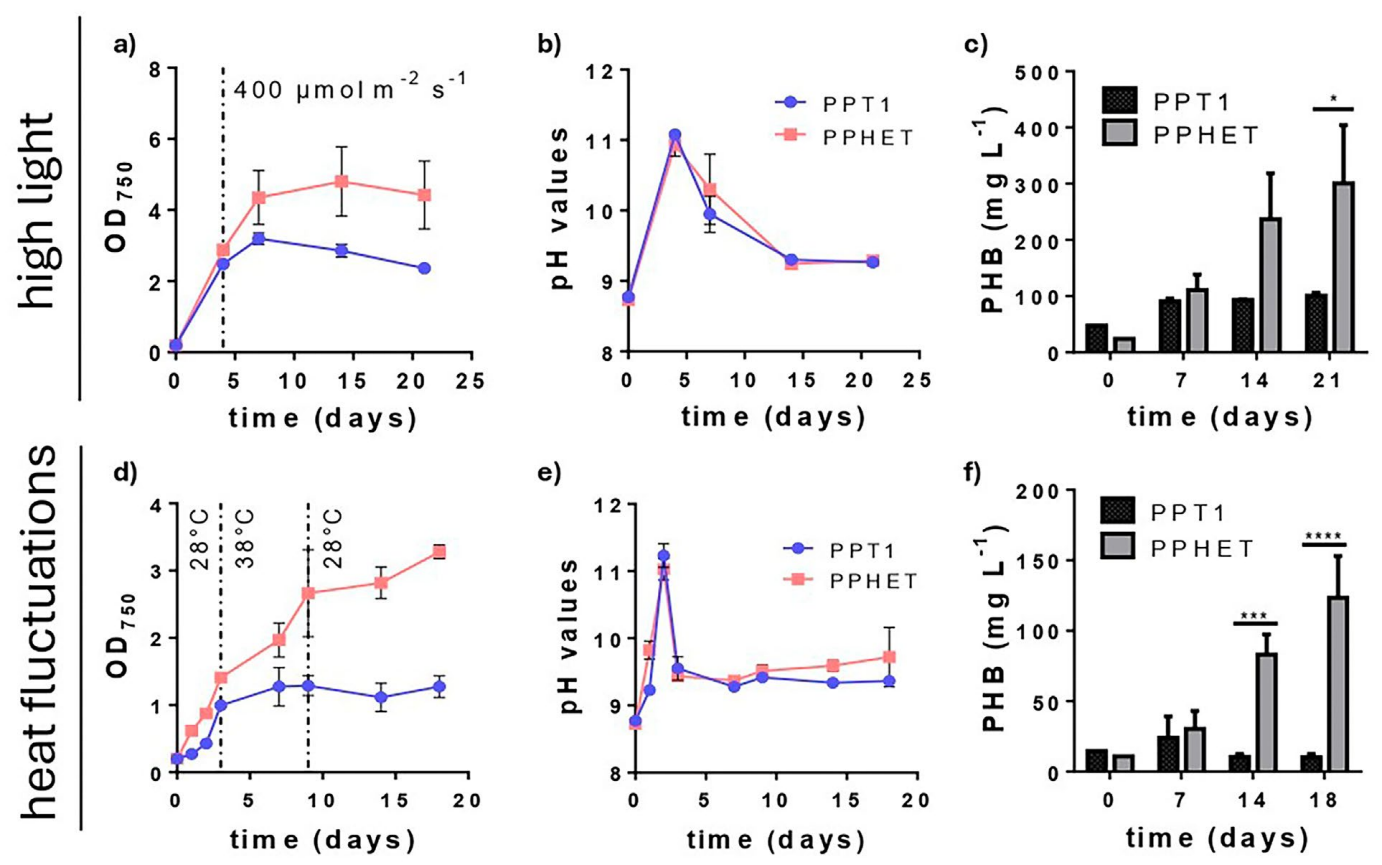
Improved robustness of PPHET compared to PPT1. Comparison of PPHET (pink) and PPT1 (blue) under high light (top) and heat fluctuations (bottom). (a, d) Growth curve. Dashed lines indicate the point at which 400 μmol m^−2^ s^−1^ was reached (a) and when the temperature was shifted (d). (b, e) pH behaviour over time. (c, f) PHB production evaluated in mg L^−1^. Data points and column bars represent the mean of at least two biological replicates at each time point ±SD. For PHB data analysis, statistical significance was determined using two‐way ANOVA followed by Sidak's multiple comparisons test. Only statistically significant differences are indicated. These observations are further verified in big scale in the next section.

To evaluate the effect that abiotic factors have on culture fitness, the experiments were conducted under sterile conditions. PPT1 and PPHET were cultivated in BG11_0_ medium supplemented with 4 mM NaNO_3_ and 10 mM NaHCO_3_, without any carbon boost upon nitrogen depletion (Figure S3). To determine the effect of high light stress, light intensity was gradually increased to 400 μmol m^−2^ s^−1^. Under these conditions, both systems exhibited similar pH trends (Figure 3b), but their physiological responses diverged. PPT1 growth was strongly inhibited under high light, with OD_750_ decreasing to ~2 by day 21 and PHB production limited to ~100 mg L^−1^ (Figure 3a,c). In contrast, PPHET maintained pigmentation and stable biomass (OD_750_~5) with PHB titers averaging ~300 mg L^−1^, indicating improved stress tolerance and continued carbon flux towards PHB synthesis (Figure 3c). We hypothesised that this effect could be due to excess oxygen accumulation in the medium caused by an overstimulated photosynthesis, potentially affecting culture robustness. Consistent with this hypothesis, qualitative bioreactor observations indicate that heterotrophic members may limit oxygen accumulation and related growth impairment (Supporting Information S3).

To assess thermal tolerance, cultures were exposed to a transient heat shock (38°C) followed by a return to optimal conditions. PPT1 exhibited irreversible bleaching and growth arrest, with no recovery upon temperature normalisation (Figure 3a; Figure S3). In contrast, PPHET maintained pigmentation during heat stress and resumed growth upon return to standard conditions. PHB production remained detectable in PPHET (up to 150 mg L^−1^), whereas it was negligible in PPT1 (Figure 3c).

These results suggest that PPHET confers resilience to high light intensities and transient heat stress and supports functional recovery.

### 4.5 Metabolic Flexibility of the Hybrid Microbiome

Upscaling PHB production in axenic cyanobacterial cultures is limited not only by poor robustness but also by the requirement for chlorosis as a trigger for PHB accumulation. Chlorosis is induced by nitrogen depletion combined with the presence of sufficient light (Klotz et al. 2015), a condition that is difficult to achieve uniformly at scale, particularly in large reactors where the surface‐to‐volume ratio limits effective illumination. Without it, cultures fail to enter chlorosis and do not accumulate PHB.

According to our microscopic results, the two microbiomes not only rely on their cyanobacteria population for PHB production, but also on other members of the community (Figure 1e). Assuming that the PPHET community is composed exclusively of *Synechocystis* PPT1, we concluded that the other members of the community that are stained with BODIPY in Figure 1e (indicated by black arrowheads), are non‐cyanobacterial species, likely capable of a heterotrophic metabolism. Supporting this view, Altamira‐Algarra and colleagues (Altamira‐Algarra et al. 2024), showed that in the WCRM, PHB production can be induced by first allowing photoautotrophic growth and then providing sodium acetate under dark incubation conditions. We reasoned that a similar approach might also work in PPHET.

**FIGURE 4:**
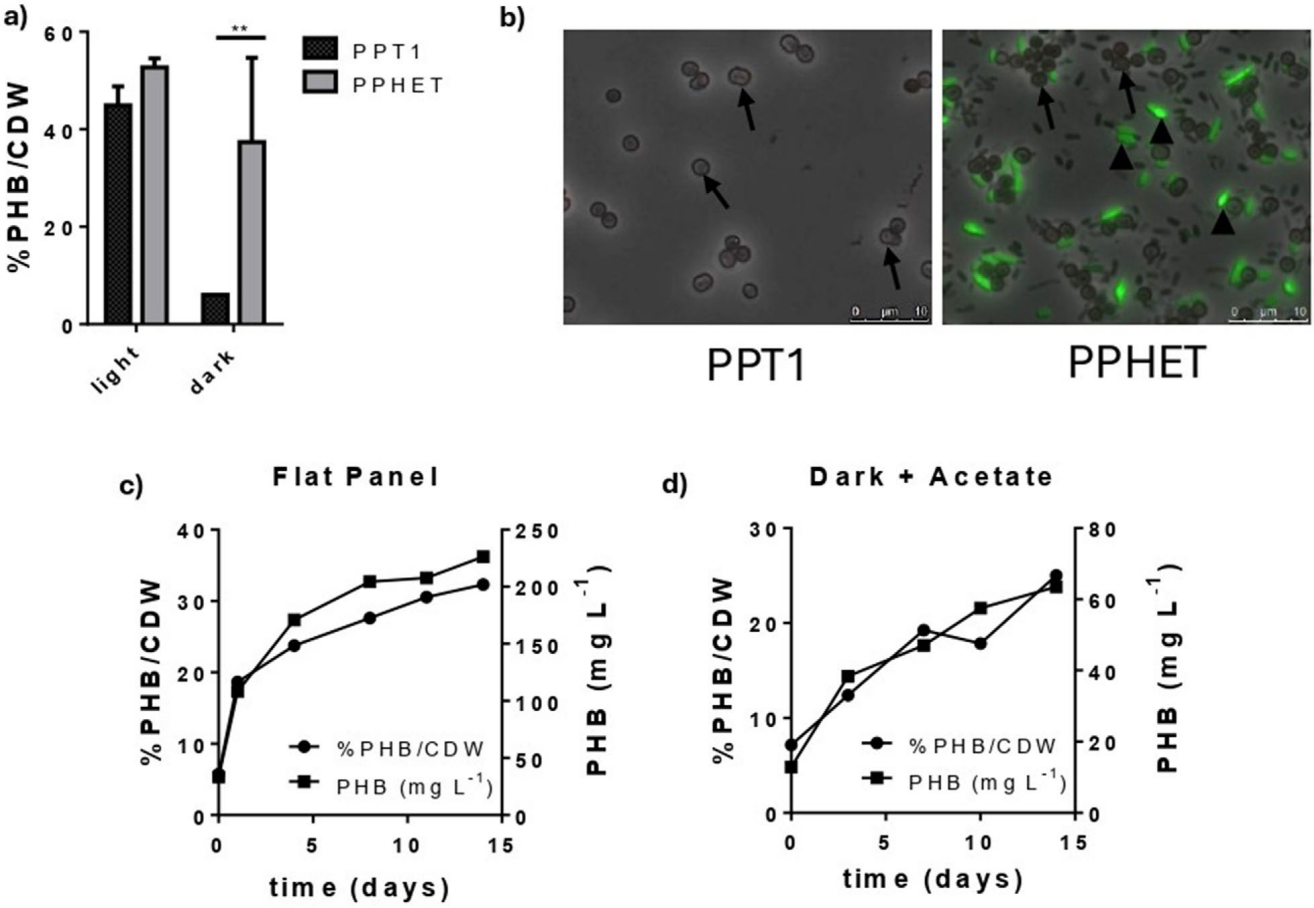
Versatility of PHB production with PPHET and upscale scenario for industrial use. (a) PHB content in PPT1 and PPHET communities measured two weeks after 30 mM sodium acetate supplementation under light and dark conditions in small‐scale cultures. Column bars represent the mean of three biological replicates ±SD. Statistical significance was determined using two‐way ANOVA followed by Sidak's multiple comparisons test. Only statistically significant differences are indicated. (b) Fluorescence microscopy images of PPT1 and PPHET following acetate‐induced PHB production in darkness. PHB granules were visualised by BODIPY staining (GFP channel) and overlaid with phase‐contrast images. Arrows indicate PPT1 cells, in which no stained granules are detected in either axenic or hybrid cultures. Black arrowheads highlight non‐cyanobacterial cells that contain BODIPY‐stained PHB granules in PPHET. Images show one representative of the three biological replicates per condition. (c and d) Time‐course of PHB accumulation in the flat‐panel photobioreactor system under nitrogen‐limited, high‐light conditions (c) and in the fermenter under dark conditions with acetate supplementation (d). Data are presented as %PHB/CDW and total PHB titre (mg L^−1^), each dot represents a single biological replicate.

To test this, we applied a heterotrophic induction strategy to PPHET. Following photoautotrophic growth, cultures were transferred to BG11_0_ supplemented with 30 mM acetate and 10 mM NaHCO_3_ and incubated under either continuous light (as a control) or complete darkness. Under light conditions, PPHET achieved ~55% PHB/CDW compared to ~45% in PPT1. In darkness, only PPHET continued to accumulate PHB, reaching ~38% PHB/CDW, whereas in PPT1, PHB showed no further increase (Figure 4a). Microscopy confirmed that in dark‐grown PPHET, PHB granules localised primarily in heterotrophic members of the community (indicated by black arrowheads in Figure 4b), rather than in PPT1 cells (indicated with black arrows in Figure 4b). Interestingly, not all non‐cyanobacterial members contributed equally to the final PHB production. It is worth mentioning that the overall biodiversity and PHB accumulation differ consistently in Figure 1e and Figure 4b, indicating that while photoautotrophic and fermentative conditions both enable PHB accumulation, they shape the PPHET microbiome differently.

Absolute PHB titers also varied between conditions due to biomass differences. Light‐incubated cultures reached OD_750_ ~3 (~250 mg L^−1^ PHB in PPHET), whereas in darkness, cultures remained at OD_750_~1 (~23 mg L^−1^ PHB) (Figure S4a,b). This reflects the fact that nitrogen‐depleted cyanobacterial cultures undergoing chlorosis complete a final round of cell division before entering dormancy, while in darkness, cultures did not undergo chlorosis and biomass accumulation ceased.

### 4.6 Improved Scalability of Hybrid Microbiomes Under Pre‐Industrial Conditions

To further confirm our observations under more realistic conditions and to address the limitations of having only duplicates in the “high‐light” experiment, we validated our results under pre‐industrial, non‐sterile conditions. For this purpose, both the axenic *Synechocystis* strain PPT1 and the hybrid PPHET community were cultivated in parallel. The experiment was divided into two phases: (i) a growth phase in 10 L glass vessels, and (ii) a PHB production phase, induced either photoautotrophically via chlorosis in flat‐panel reactors with high surface‐to‐volume ratio (to ensure sufficient light supply) or heterotrophically in dark glass reactors wrapped with aluminium foil and supplemented with acetate. Phase (ii) was inoculated directly with cultures from phase (i).

PPHET and the axenic PPT1 were grown non‐sterile under identical conditions, starting with BG11_0_ supplemented with 5 mM NaNO_3_ and 10 mM NaHCO_3_. Cultures were initially illuminated on one side at an intensity of 100 μmol m^−2^ s^−1^. At first, both systems tolerated non‐sterile conditions and reached OD_750_ ~1. At this point, an additional side illumination of 100 μmol m^−2^ s^−1^ was applied to promote further biomass accumulation and nitrate consumption. This led to immediate PPT1 collapse, followed by biomass loss and complete bleaching (Figure S5c). This result confirms observations from small‐scale cultivation, where increased light intensity impaired growth and PHB production in PPT1, demonstrating the consistency of our findings in a different setup. Contamination plating revealed the presence of microbial contaminants in PPT1 (Figure S5d); however, based on small‐scale experiments, non‐sterility did not appear to limit performance (Figure S2). We therefore assume that these contaminants are unlikely to be the primary cause of culture collapse and represent instead a secondary burden. In contrast, PPHET continued to grow, even after an additional side illumination of 100 μmol m^−2^ s^−1^, reaching OD_750_~4 after 29 days (Figure S5a,b). These results emphasise the enhanced resilience of PPHET under scale‐up conditions.

We further validated PHB production in scaled systems. Given the challenges of inducing full chlorosis in large, high‐density cultures due to light limitations, PPHET was transferred into a flat‐panel photobioreactor (4 L volume, 3 cm light path) and exposed to 300 μmol m^−2^ s^−1^ light in the presence of NaHCO_3_. Under these conditions, PPHET reached 33% PHB/CDW (~250 mg L^−1^) (Figure 4c). A separate dark‐scale fermentation using an 8 L, non‐aerated, foil‐wrapped reactor supplemented with acetate yielded 22% PHB/CDW (~70 mg L^−1^) (Figure 4d). Comparative analysis revealed that while PPT1 could accumulate PHB under controlled, small‐scale conditions, it consistently failed under scale‐up stress, unlike the robust and flexible PPHET system.

## 5 Discussion

As a general conclusion, these results demonstrate the superior robustness and versatility of a hybrid microbiome (PPHET) compared to axenic cultures under multiple stress conditions and scaling regimes. Here, we discuss the ecological and biotechnological implications of these findings, as well as potential mechanisms underlying the enhanced performance of PPHET.

### 5.1 Community Composition and Functional Resilience of Cyanobacterial Consortia

Next‐generation sequencing (NGS) analysis of the WCRM and PPHET communities revealed taxonomic profiles resembling those of natural cyanobacteria‐dominated ecosystems. Such systems exhibit conserved community patterns and recurring keystone taxa (Kust et al. 2025), which have been extensively characterised and provide a robust ecological framework for interpreting our results. Both consortia harboured members of the order Rhodobacterales, Flavobacteriales, Rhizobiales, and Sphingomonadales, taxa commonly associated with biogeochemical cycling and symbiotic interactions (Altamira‐Algarra, Sun, et al. 2025; Garcia‐Pichel 2023; Kust et al. 2025). These groups commonly co‐occur with cyanobacteria in diverse environments such as in freshwaters (Kust et al. 2025), biological soil crusts (Garcia‐Pichel 2023; Nelson et al. 2021; Van Goethem et al. 2017), marine systems (Mutalipassi et al. 2021), and lichen symbioses (Aschenbrenner et al. 2016). In such communities, cyanobacteria act as primary producers, fixing carbon in nutrient‐poor environments and thereby supporting the growth of other community members (Garcia‐Pichel 2023; Kust et al. 2025). These communities also include organisms capable of anoxygenic photosynthesis, such as Rhodobacterales and Sphingomonadales, suggesting that light‐driven energy production is a major metabolic strategy for cyanobacteria‐associated bacteria, with electron donors likely derived from organic substrates (Duxbury et al. 2025; Kust et al. 2025). Symbiotic relationships that facilitate the production of essential metabolites further supporting community stability and function, have also been described (Duxbury et al. 2025). In such systems, heterotrophic members consume oxygen, preventing the buildup of reactive oxygen species (ROS) and photorespiration, both of which inhibit cyanobacterial growth (Ku and Edwards 1977). Moreover, heterotrophs are inherently more resilient to oxidative stress due to their robust ROS‐scavenging systems, in contrast to cyanobacteria (Sandrini et al. 2020; Szeinbaum et al. 2021). These findings highlight how ecological principles governing the dynamics of natural microbial consortia can be leveraged to engineer communities with enhanced functionality and resilience for biotechnological applications (Giri et al. 2020).

### 5.2 Robustness of the Microbiome Against Abiotic Stress Factors

Fluctuations in light intensity and temperature represent major challenges for stable cultivation in industrial photobioreactors, often impairing cyanobacterial growth and productivity (Amin et al. 2024; Wahal and Viamajala 2010). In this study, the axenic strain PPT1 exhibited sensitivity to high light, which may be caused by overstimulated photosynthesis leading to excessive oxygen production. This condition promotes photorespiration and the generation of reactive oxygen species (ROS), resulting in oxidative stress and reduced metabolic efficiency (Apel and Hirt 2004; Ku and Edwards 1977). In contrast, the synthetic community PPHET demonstrated enhanced resilience, maintaining stable growth and PHB production. A possible explanation for this robustness is the presence of heterotrophic partners that scavenge excess oxygen, mitigating oxidative stress, a phenomenon previously reported in microalgae‐associated consortia (Krohn et al. 2022).

Similarly, PPHET outperformed PPT1 under elevated temperature conditions, further supporting the protective role of the heterotrophic community in buffering environmental stress (Awasthi et al. 2014). These findings highlight the potential of consortium‐based systems for large‐scale applications, where environmental fluctuations are inevitable. Overall, the enhanced resilience of PPHET aligns with the broader ecological principle that biodiversity confers stability and functional conservation under abiotic stress (Awasthi et al. 2014; Giri et al. 2020).

### 5.3 Metabolic Versatility in PHB Production Pathways With Hybrid Microbiomes

Nitrogen limitation is one of the most investigated conditions for PHB accumulation in cyanobacteria, typically implemented via a two‐step cultivation process involving biomass generation followed by nitrogen starvation in a separate reactor (Forchhammer and Schwarz 2019). However, effective induction of chlorosis and PHB biosynthesis requires sufficient light to activate redox‐based signalling pathways (Klotz et al. 2015), posing a significant limitation for large‐scale systems due to poor light penetration and high infrastructure demands (Price et al. 2022).

In this study, we demonstrate the metabolic adaptability of PPHET, providing potential strategies better suited for industrial scalability. First, flat‐panel photobioreactors with high surface‐area illumination enabled effective chlorosis induction and PHB accumulation after nitrate depletion. This design is amenable to solar‐driven systems, reducing operational complexity and costs, consistent with previous work using sunlit or open cultivation platforms (Carone et al. 2024; Price 2022). Second, we show that PHB synthesis can also be achieved under dark conditions via acetate supplementation. This reflects fermentative PHB production by heterotrophic members of the community, providing an alternative to light‐dependent, photoautotrophic accumulation. A similar dark‐phase production strategy has been validated using the natural WCRM microbiome, supporting the viability of alternating light and dark cultivation modalities (Altamira‐Algarra et al. 2024).

These findings underscore the value of hybrid microbiomes in enhancing operational flexibility. The ability to switch between photoautotrophic and heterotrophic production modes allows adaptation to variable cultivation environments and simplifies process control. Given the challenges of sterile, tightly regulated phototrophic cultivation (Price et al. 2022; Rueda et al. 2023; Schmelling and Bross 2024), microbiome‐based systems may offer a promising approach for scalable and potentially more economically viable bioproduction (Altamira‐Algarra, Garcia, and Gonzalez‐Flo 2025).

### 5.4 PHB Quantification and Productivity

In this study, we quantified PHB accumulation across different cultivation setups using multiple metrics: %PHB per cell dry weight (CDW), total PHB titre (mg L^−1^), and volumetric productivity (mg L^−1^ day^−1^). The primary purpose of reporting these values is not to establish absolute performance benchmarks, but to demonstrate the functional flexibility and enhanced robustness of the hybrid microbiome under diverse operational conditions.

PPHET achieved the highest PHB accumulation at small scale, reaching approximately 600 mg L^−1^. This corresponds to an overall productivity of ~35 mg L^−1^ day^−1^, when accounting for both growth and the 14‐day chlorosis phase, and ~42 mg L^−1^ day^−1^ when considering only the chlorosis phase. For comparison, chlorosis‐phase productivity in the upscaled systems was ~18 mg L^−1^ day^−1^ in the flat‐panel reactor (250 mg L^−1^ final yield) and ~5 mg L^−1^ day^−1^ in the fermentative setup (70 mg L^−1^). These results highlight that small‐scale outcomes do not directly translate to larger systems, emphasising the need for reactor‐specific performance evaluations. Nevertheless, scale‐up optimisation was beyond the scope of this study and represents an important avenue for future research.

## 6 Conclusions

This study provides proof‐of‐concept evidence that a natural, complex microbiome can stably host genetically engineered cyanobacteria for phototrophic PHB production. By harnessing the ecological benefits of microbial consortia, this approach aims at alleviating key limitations associated with strictly axenic cultivation and offers a promising framework for upscaling scenarios. Further optimisation will be required to enhance overall productivity, including strategies to increase PHB yield per biomass and improve bioreactor design on a large scale. In addition, future studies incorporating temporal analyses of community composition will be essential to better understand consortium dynamics and their influence on production performance. Together, these findings show the potential of microbiome‐based approaches to advance cyanobacterial biotechnology and support the development of resilient product production platforms.

## Author Contributions

**Arianna Zini:** conceptualization (equal), investigation (lead), writing – original draft (lead), methodology (lead), validation (lead), visualization (lead), writing – review and editing (equal), data curation (lead), formal analysis (lead), project administration (lead). **Jennifer Müller:** formal analysis (16S rRNA sequence analysis), writing – original draft (16S library preparation and sequence analysis), writing – review and editing (supporting). **Phillipp Fink:** investigation (HPLC measurements), writing – review and editing (supporting). **Karl Forchhammer:** conceptualization (equal), supervision (lead), funding acquisition (lead), resources (lead), writing – review and editing (lead).

## Funding

This work was supported by Bundesministerium für Bildung und Forschung (031B1399B).

## Conflicts of Interest

The authors declare no conflicts of interest.

## Supporting information


**Data S1:** mbt270302‐sup‐0001‐Supinfo.pdf.

## Data Availability

The data for this study have been deposited in the European Nucleotide Archive (ENA) at EMBL‐EBI under accession number PRJEB98235 (https://www.ebi.ac.uk/ena/browser/view/PRJEB98235).
