# Supplementary material for: Cultivation in a Natural Microbial Community Enhances the Industrial Performance of a Genetically Engineered Cyanobacterium for Bioplastic Production
Source: Microb Biotechnol. 2026 Jan 20;19(1):e70302. doi: 10.1111/1751-7915.70302 (PMC12819161; doi:10.1111/1751-7915.70302)
Supplement: Supplementary file 1 — Data S1: mbt270302‐sup‐0001‐Supinfo.pdf. [file MBT2-19-e70302-s001.pdf]

Supporting information

Table 1. List of the cultures used in this study.

NAME	GENOTYPE	REFERENCE
PPT1	*Synechocystis* sp. PCC 6803, KanR, GenR	Koch *et al.*, 2020
GFP	*Synechocystis* sp. PCC 6803, SpecR	Orthwein *et al.*, 2021
WRCM	Wild Cyanobacteria Rich Microbiome	Altamira-Algarra *et al.*, 2024
PPHET	PPT1 with non-cyanobacterial part of WCRM	This study

Table 2. List of the microorganisms grouped in the group "Others" in Fig. 2b, with relative abundancy lower than 0,05% at the time of sampling.

PHYLUM	CLASS	ORDER	NOTES
Actinobacteriota	Actinobacteria	Micrococcales	
Myxococcota	Myxococcia	Myxococcales	
Proteobacteria	Gammaproteobacteria	Enterobacterales	
Proteobacteria	Gammaproteobacteria	Burkholderiales	
Cyanobacteria	Cyanobacteriia	Phormidesmiales	present only in WCRM
Acidobacteriota	Acidobacteriae	Bryobacterales	
Cyanobacteria	Cyanobacteriia	Leptolyngbyales	present only in WCRM
Verrucomicrobiota	Verrucomicrobiae	Chthoniobacterales	present only in WCRM

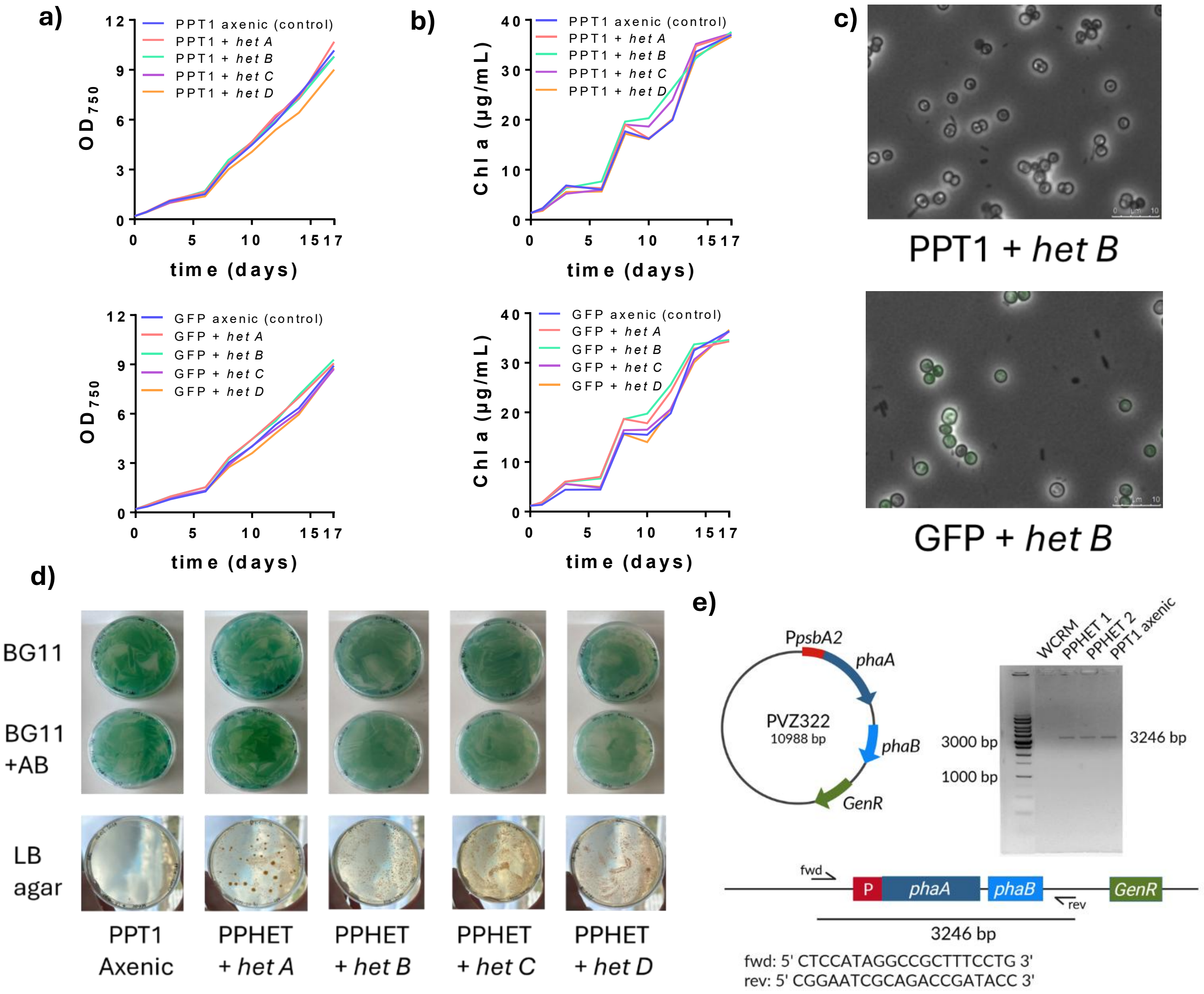

Figure S1: Hybrid microbiome establishment and stability. a-b) Growth (OD_{750}) and chlorophyll *a* accumulation were monitored over time to specifically track the phototrophic component (cyanobacteria) within the synthetic communities; **b)** Microscopic visualization of the new microbiomes. Images show overlays of phase-contrast and GFP fluorescence channels to enable simultaneous observation of overall cellular morphology and on the right, the distribution of GFP-expressing cyanobacteria within the community; **c)** To confirm that PPT1 remained the dominant phototroph within the community, cultures were plated on BG11 agar both with and without the addition of antibiotics to which PPT1 is resistant (Kanamycin, specifically). Growth under selective conditions confirmed stable integration of the engineered cyanobacterium into the microbiome. Community structure was qualitatively assessed by plating on nutrient-rich medium (LB) to evaluate heterotrophic fraction; **d)** To assess long-term community stability, cultures were propagated through multiple 14-day cycles, each followed by a 1:10 dilution into fresh BG11 medium. **e)** To verify the continued presence of the engineered strain and exclude residual presence and overgrowth by wild-type *Synechocystis*, colony PCR targeting the PPT1 plasmid was performed at multiple time points. The plasmid is reported with the Promotor *psbA2* (P*psbA2*) from *Synechocystis* and *phaAB* gene cassette from *Cupriavidus necator* H16, which were assembled in the PVZ322 vector (Koch *et al,* 2020). The linearized plasmid is represented at the bottom of the picture, and semi-arrows correspond to primers annealing to it (primer sequences reported). No amplification was observed in the wild-type community (WRCM), while distinct bands corresponding to *phaA* and *phaB* were detected in the PPHET community after initial inoculation (PPHET 1) and after the second propagation cycle (PPHET 2). A positive control from the axenic PPT1 culture is also shown. This result confirms successful integration of the engineered strain PPT1 within the hybrid community.

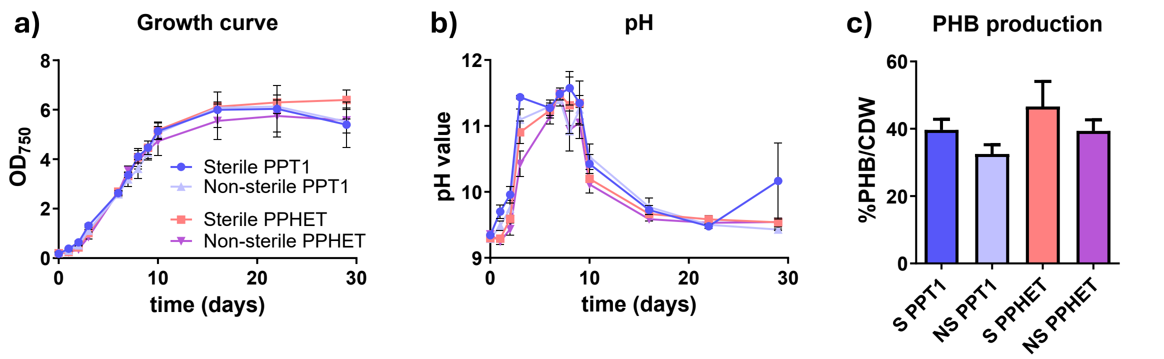

Figure S2: PPT1 and PPHET in sterile (S) and non-sterile (NS) conditions do not show major differences. Cultures were cultivated in 50 mL Erlenmeyer flasks with the standard parameters of temperature, light and mixing. The medium used was $BG11_0$ supplemented with 10 mM $NaHCO_3$ and 5 mM $NaNO_3$. At day 8, 10 mM $NaHCO_3$ were added. The experiment was run for 29 days in total. Data points and column bars represent the mean of three biological replicates ±SD. **a)** Growth curve measured at OD_{750}. **b)** pH monitoring. **c)** PHB in %PHB/CDW of PPT1 and PPHET at the end of the production process.

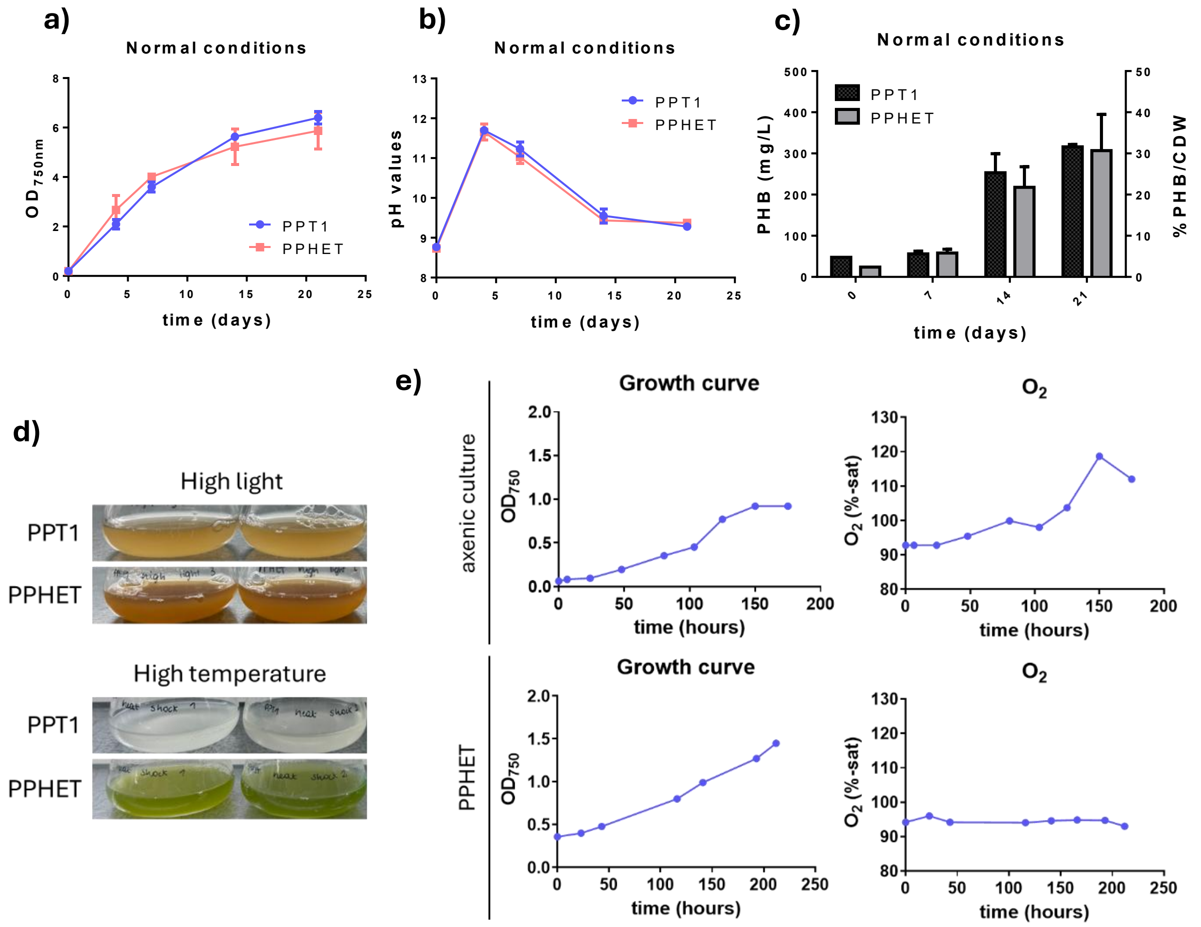

Figure S3: Effects of abiotic stress factors on culture fitness. Normal conditions as a control to the abiotic stress test do not show big differences. **a)** Growth curve measured at OD_{750}. **b)** pH monitoring. **c)** PHB production of PPT1 and PPHET over time. **d)** Pictures of the cultures PPT1 and PPHET (duplicates) taken at the end of each experiment under high light (left) and under "heat shock" (right). **e)** Qualitative observations consistent with the hypothesis that heterotrophic members may counteract oxygen accumulation in the medium and reduce oxidative stress. This analysis is based on a single bioreactor run using a setup different from those employed in the main experiments and is therefore intended as qualitative support rather than quantitative validation.

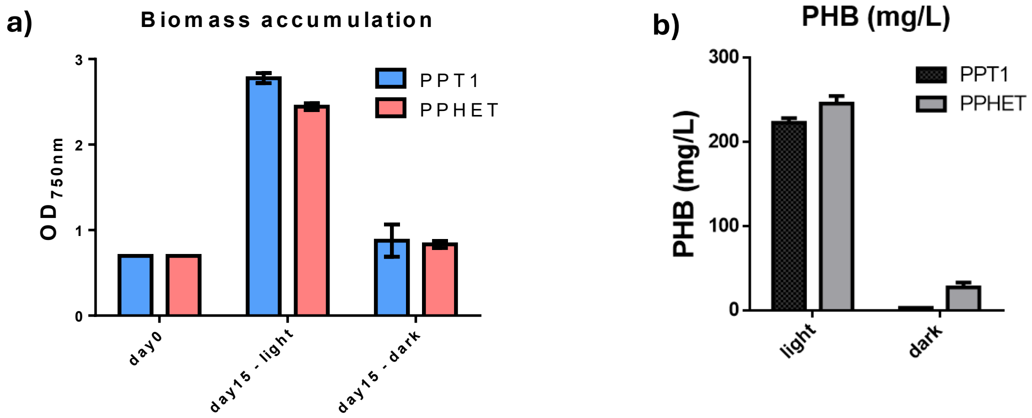

Figure S4: The biomass accumulation influences the final PHB titer. Cultures were analysed after 14 days upon addition of acetate, both under light and dark conditions. **a)** Biomass accumulation in OD_{750}. **b)** PHB production in terms of mg/L.

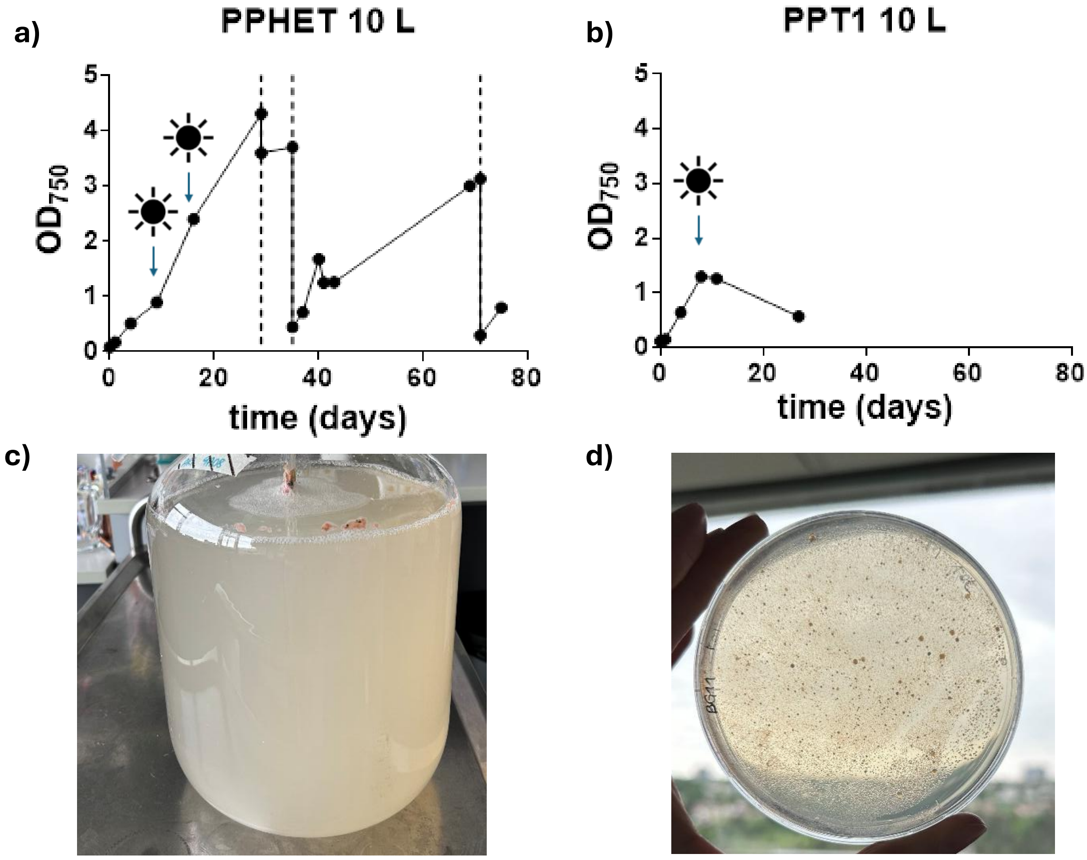

Figure S5: Abiotic perturbations in large volumes cause the collapse of the axenic PPT1 culture. **a)** Long-term cultivation of the PPHET consortium in a 10 L photobioreactor over ~80 days. Optical density (OD_{750}) was monitored to assess growth dynamics. Sun symbols indicate steps of increased light intensity. Vertical dotted lines in the graphs indicate the time points at which the cultures completely consumed the initial nitrate supply, marking nitrate depletion events. These were followed by partial harvesting of the cultures and replenishment with fresh $BG11_0$ medium supplemented with 1 to 4 mM $NaNO_3$. **b)** Growth profile of axenic PPT1 strain in a 10 L photobioreactor. A sharp decline in biomass was observed following increased light intensity, indicating light-induced culture collapse. **c)** Collapsed PPT1 culture in the 10 L vessel. **d)** Growth of the collapsed culture on solid media to check for contamination.

a) b)

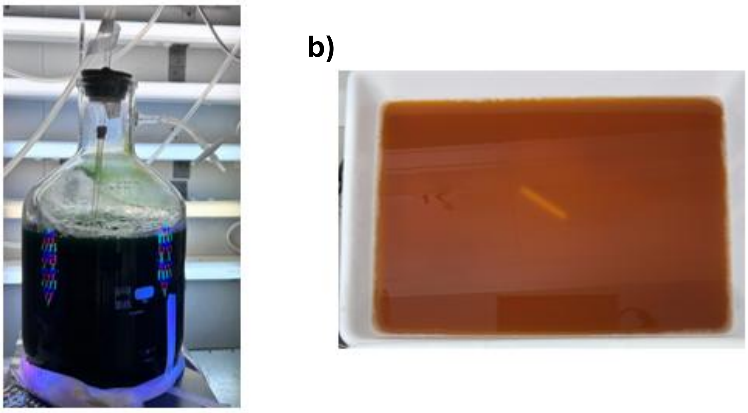

Figure S6: Successful scaling up attempts with PPHET for growth and PHB production. a) PPHET in the 10 L flask for normal growth. **b)** Flat Panel reactor with a 4 L volume at the end of the production process.
